# Injectable Hyaluronic Acid and Amino Acids Complex for Pediatric Hard-to-Heal Wounds: A Prospective Case Series and Therapeutic Protocol

**DOI:** 10.3390/children12111554

**Published:** 2025-11-17

**Authors:** Guido Ciprandi, Biagio Nicolosi, Gabriele Storti, Simone F. Marino, Carlotta Scarpa, Franco Bassetto

**Affiliations:** 1 Pediatric Wound Care MasterClass Foundation, 00050 Rome, Italy; 2 Department of Health Professions, Meyer Children’s Hospital IRCCS, 50139 Florence, Italy; biagio.nicolosi@meyer.it; 3 Unit of Plastic Surgery, Department of Surgical Sciences, University of Rome “Tor Vergata”, 00133 Rome, Italy; gabriele.storti@uniroma2.it; 4 Division of Plastic and Maxillofacial Surgery, Bambino Gesu’ Children’s Hospital IRCCS, 00050 Rome, Italy; simone.marino@opbg.net; 5 Complex Unit of Plastic Surgery, University of Padua, 35122 Padua, Italy; carlotta.scarpa@unipd.it (C.S.); franco.bassetto@unipd.it (F.B.)

**Keywords:** wounds, long-delayed wound healing, children, adolescent, hyaluronic acid, amino acids, injectable complex, tissue repair-regeneration

## Abstract

**Highlights:**

**What are the main findings?**

- Complex soft-tissue injuries in pediatric patients, which may delay healing beyond six months due to a recalcitrant, chronic, or stalled wound, can be effectively treated with an injectable therapy combining hyaluronic acid and six amino acids in a single formulation. Regardless of the etiopathogenesis, the results allow for tissue repair and regeneration up to complete re-epithelialization.

**What is the implication of the main finding?**

- The results obtained, with no pain caused by the therapy itself, show a reduction and disappearance of pain from the chronic condition after just the first two injections, and complete repair of the complex lesion within a maximum of six weeks from the start of treatment. A six-month follow-up confirms stable outcomes with no relapse.

**Abstract:**

Background: Pediatric hard-to-heal wounds are rare but clinically demanding due to skin immaturity, comorbidities, and infection risk. Methods: This prospective case series evaluated the feasibility, safety, and clinical outcomes of an injectable hyaluronic acid-amino acid complex administered to fifteen children and adolescents (aged 4–16 years) with chronic hard-to-heal wounds, treated between November 2022 and August 2025 within a standardized wound-hygiene protocol. The primary outcome was time to complete re-epithelialization; secondary outcomes included pain, tolerability, and safety. Results: Complete healing was achieved in most patients within a few weeks of treatment. The injectable therapy was well tolerated, with minimal discomfort and no serious adverse events observed. Conclusions: The injectable hyaluronic acid-amino acid complex appears to be a safe, feasible, and potentially effective therapy for pediatric hard-to-heal wounds. These preliminary findings support its integration into multidisciplinary wound-care strategies, although controlled multicenter studies are warranted to confirm efficacy and define optimal protocols.

## 1. Introduction

### 1.1. Defining Hard-to-Heal Wounds in Pediatric Patients

Hard-to-heal wounds are commonly defined as those that, despite appropriate clinical management, fail to reduce in area by more than 30% within four weeks and do not achieve complete closure within 12 weeks [1,2]. These thresholds, however, are primarily derived from the adult population and are not fully standardized for pediatrics [3,4,5]. In adults, different etiologies are associated with variable parameters, such as: <50% reduction in diabetic foot lesions; <40% in venous lesions of the lower extremities; 20–40% in pressure ulcers. These criteria cannot be directly applied to children and adolescents. In pediatric patients, particularly neonates and infants with immature skin and a developing immune system, the healing process is often different, and shared definitions of hard-to-heal wounds are still lacking [5,6,7,8]. This lack of unambiguous definitions underscores the importance of adopting an interdisciplinary and interprofessional approach (IIPT) to accurately identify and manage complex wounds [1]. Furthermore, growing evidence indicates that impaired or stalled healing in children is particularly associated with the presence of multidrug-resistant polymicrobial biofilms, responsible for 80% of chronic wounds. However, host factors, such as prematurity, malnutrition, and chronic diseases, also contribute to wound chronicity [9].

The lack of specific pediatric criteria for defining hard-to-heal wounds highlights the need for personalized strategies and collaborative care models.

### 1.2. The Fragile, Immature Skin in Pediatric Patients

Neonatal and infant skin, although structurally similar to adult skin, are markedly immature, characterized by a thinner stratum corneum and reduced dermo-epidermal cohesion—factors that increase susceptibility to trauma, infection, and transepidermal water loss (TEWL). The epidermis and stratum corneum are thinner, with reduced cohesion at the dermal-epidermal junction, making the skin more susceptible to trauma and TEWL. The dermis contains less organized collagen fibers and is characterized by reduced elastin synthesis, both factors that compromise resistance to friction and slippage [10].

Wound repair in pediatric age proceeds according to the physiological process that includes the inflammatory, proliferative, and remodeling phases, but obviously with unique characteristics: the inflammatory response is attenuated, while fibroblast activity and collagen deposition can occur more rapidly [11].

The immaturity of the skin barrier increases the risk of infections and fluid-electrolyte imbalance. Additional host-related factors such as nutritional deficiencies (proteins, vitamins A, C, D, iron, zinc, selenium), chronic diseases, and prematurity may further compromise the already fragile pediatric healing process [12].

These unique structural and functional characteristics make pediatrics’ skin more susceptible to injury and healing complications, underscoring the need for informed and specialized approaches.

Advanced dressings that maintain a balanced moist environment and support the extracellular matrix (ECM), such as Hyaluronic Acid (HA)-based formulations, can help preserve barrier function, promote cell proliferation, and reduce inflammation, thus optimizing the healing process in this particularly fragile population [10].

### 1.3. The Role and Properties of HA in Pediatric Skin

HA, the main glycosaminoglycan in the ECM, is a linear, unsulfured, and highly hydrophilic polysaccharide chain capable of binding large amounts of water due to its structure rich in carboxyl and acetylamine groups (hygroscopic properties). This characteristic gives the skin remarkable turgidity and elasticity and promotes the diffusion of nutrients, growth factors, and immune cells.

Under physiological conditions, HA is completely dissociated, negatively charged, and participates in numerous key biological processes, including cell proliferation and differentiation, tissue remodeling, and modulation of the inflammatory response [13,14].

Unlike other glycosaminoglycans, HA is unsulfured and is synthesized directly at the plasma membrane by three isoforms of hyaluronan synthase (HAS1–S3), which determine its different molecular weights. Its degradation is regulated by hyaluronidase isoforms, with a particularly rapid turnover in childhood, related to higher metabolic rates and skin growth and maturation processes. Pediatrics’ skin, despite representing approximately 15% of body weight, contains more than half of the total HA present in the body: while an adult has an average of 10–15 g of HA, a 1-year-old child has approximately 2 g, a 5-year-old approximately 4 g, and a 10-year-old up to 6 g [15,16].

The cutaneous distribution of HA varies between the epidermis and the dermis. In the dermis, HA, together with proteoglycans and collagen, contributes to maintaining hydration and biomechanical properties, forming a three-dimensional scaffold with marked viscoelasticity. In the epidermis, present mainly between the basal and spiny cells, HA is crucial for the organization of the stratum corneum and the formation of the lamellar structures that ensure the integrity of the skin barrier. Furthermore, by binding to specific receptors (including CD44, RHAMM, TLRs, and HARE), HA participates in cell signaling processes and exerts an antioxidant role, thus limiting damage from reactive oxygen species [14,15,16].

These properties make HA an essential component in pediatric skin physiology, suggesting a potential therapeutic role in Hard-to-Heal wounds, where reduced skin maturity and increased tissue turnover can hinder spontaneous healing [14].

Previous studies have demonstrated that topical formulations of Hyaluronic Acid combined with Amino Acids (HA+AA) promote epithelial regeneration and improve healing quality. However, evidence regarding injectable use, especially in pediatric hard-to-heal wounds, remains scarce [14].

Building on pediatric and adult data showing that topical HA+AA supports epithelial regeneration and scar quality, it was hypothesized that direct intradermal delivery could amplify these effects by concentrating substrates and signaling at the advancing edge of hard-to-heal wounds [14].

### 1.4. Rationale for the Injectable Route

Beyond topical formulations, intradermal delivery of HA combined with AA can directly target the dermo-epidermal junction and the subcutaneous plane, where fibroblasts, endothelial cells, and ECM turnover are most active in hard-to-heal wounds. Local depots may serve several purposes: (1) to concentrate substrates for collagen and elastin synthesis at the advancing edge, (2) to modulate inflammation within the microenvironment, and (3) to support angiogenesis and granulation while preserving a moist interface. This mechanistic rationale supports testing injectable HA+AA as an adjunct in pediatric wounds characterized by tissue loss, undermining, or stalled edges.

Therefore, the present prospective case series aimed to evaluate the feasibility, safety, and clinical outcomes of injectable Hyaluronic Acid and six Amino Acids (HA+6AA) in pediatric patients with hard-to-heal wounds.

## 2. Materials and Methods

### 2.1. Study Design and Setting

This was a prospective, descriptive, observational case series conducted at a single Italian pediatric wound care center. The study was explicitly designed as exploratory and hypothesis-generating, aiming to assess the feasibility, outcomes, and tolerability of injectable Hyaluronic Acid and Amino Acids (HA+AA) therapy in children and adolescents with hard-to-heal wounds. All consecutive pediatric patients meeting eligibility criteria were screened and enrolled between November 2022 and August 2025, including follow-up time.

Given the exploratory nature and rarity of the target population, as well as the ethical implications of withholding potentially beneficial treatment in children and adolescents with chronic refractory wounds, no control or comparator group was included. The study was carried out in an outpatient, multidisciplinary wound-care setting by a team composed of a pediatric physician and a wound-care nurse specialist trained in advanced wound management. The study adhered to the STROBE guidelines for observational research.

### 2.2. Patient Population and Inclusion Criteria

Eligibility was confirmed through a two-step clinical and nursing review based on predefined inclusion and exclusion criteria. Children and adolescents aged between 4 and 16 years were eligible if they presented with at least one hard-to-heal wound, defined as less than 30% reduction in wound area after four weeks of appropriate care and failure to achieve closure after more than six months from onset. Different wound types (Pressure Ulcers—PUs, surgical, traumatic, and Lower Leg Ulcer—LLU) were included to represent the full clinical spectrum of pediatric hard-to-heal lesions commonly managed in tertiary centers, provided they met the chronicity criteria defined above. Wounds were staged according to the EPUAP/NPIAP/PPPIA 2025 Guidelines [17], and written informed consent was obtained from parents or legal guardians, with child assent when appropriate.

Exclusion criteria included uncontrolled systemic or local infection requiring urgent surgical intervention, severe immunosuppression or ongoing chemotherapy or radiotherapy, uncorrected coagulation disorders, inability to complete the outpatient protocol due to logistic or family reasons and known hypersensitivity to HA+AA components.

All patients presented with severe, painful, and chronic wounds that had failed to heal despite multiple previous treatment attempts in other centers. Inclusion and exclusion criteria are summarized in Table 1.

Each patient was referred to our outpatient clinic due to delayed healing, persistent discomfort, or caregiver exhaustion. Pain was assessed using validated age-appropriate scales: the Face, Legs, Activity, Cry, Consolability (FLACC) scale [18], the Visual Analogue Scale (VAS) [19], or the Individualized Numeric Rating Scale (INRS) [20] for children with cognitive impairment. When multiple wounds were present in the same patient, an index wound was selected for analysis to avoid clustering, and all outcomes were analyzed on a per-patient basis.

### 2.3. Intervention: Injectable HA+6AA Protocol

All patients were treated within a standardized wound hygiene protocol to ensure consistency of care and optimal wound-bed preparation. The protocol included gentle cleansing of the wound bed with non-ionic, no-rinse surfactant solutions, protection of the periwound area, antisepsis with 2% chlorhexidine for the surrounding skin, and application of polyhexanide (PHMB) solution to the wound bed. Advanced dressings were applied to maintain moisture balance and homeostasis.

Povidone-iodine was deliberately excluded from the protocol due to its risk of systemic iodine absorption in children [21]. PHMB and chlorhexidine were chosen as safer and more selective antiseptics with proven pediatric tolerability [22].

The therapeutic agent consisted of a sterile injectable medical device (Vulnamin^®^ Inj, Professional Dietetics, Milan, Italy) containing a low-molecular-weight sodium hyaluronate (10 mg/mL; 150–280 kDa) combined with six AA—glycine, proline, leucine, lysine, valine, and alanine—in proprietary ratios. The product was infiltrated along the wound edges and dermo-epidermal junction, and in deeper cases within the subcutaneous plane. For each session, 2–4 mL were administered, divided into 4–8 micro-depots spaced approximately 0.5–1 cm apart. Infiltration was performed with sterile 21–23 G needles under magnification to avoid vascular injury.

All children and adolescents received oral paracetamol (15 mg/kg) 30 min before the procedure, and topical lidocaine spray or cream was applied when required, particularly in patients with sensory hypersensitivity or anxiety. Sedation was not required in any case. Each session was performed in the presence of both a parent or caregiver and the clinical team to minimize distress. Treatment sessions were repeated every 7 ± 2 days until complete re-epithelialization or clinical stability was achieved. Specific co-interventions were allowed based on predefined criteria: Negative Pressure Wound Therapy (NPWT) for extensive tissue loss, heavy exudate, or undermining; and Dialkylcarbamoyl Chloride (DACC) dressings for superficial critical colonization identified according to the International Wound Infection Institute (IWII) NERDS/STONEES (Non-healing, Exudate, Red friable tissue, Debris, Smell/Size increasing, Temperature, Os, New areas of breakdown, Exudate, Erythema/Edema, Smell) criteria [23].

At each session, procedural pain, bleeding, and any local or systemic adverse events were recorded. Major safety endpoints included injection-site infection, nodule formation, delayed healing, and hypersensitivity reactions.

### 2.4. Co-Interventions. Indications and Standardization

Predefined criteria guided the use of adjunctive therapies. NPWT was indicated when ≥1 of the following applied: (1) extensive tissue loss and/or cavity/undermining judged at risk of collapse, (2) heavy exudate requiring device-level control, (3) stage IV lesions or stage III with ≥1 cm undermining, or (4) pain interfering with dressing tolerance despite optimized analgesia. NPWT was standardized to continuous negative pressure of −75 mmHg for the first 24 h, then −100 mmHg thereafter, macrofoam filler trimmed to half thickness, and dressing changes every 3–4 days. When combined with HA+AA injections, NPWT was initiated approximately 30 min after infiltration to stabilize fragile granulation and control exudate.

DACC dressings were used for clinical signs of critical superficial colonization (IWII NERDS/STONEES stage 1–2) until signs resolved, typically 7–10 days.

Topical HA+AA gel/cream was reserved for osmotic/enzyme-assisted autolysis when eschar/slough was present.

### 2.5. Outcomes and Endpoints

The primary endpoint was the time to complete re-epithelialization, defined as 100% epithelial coverage of the wound without exudate, confirmed at two consecutive assessments at least 7–14 days apart.

Secondary endpoints included percentage reduction in wound area at four weeks, time to 50% wound-area reduction, pain intensity (assessed with FLACC, INRS, or VAS depending on age and cognitive status), exudate quantity and odor (rated on a 0–3 ordinal scale), presence of local infection based on the IWII NERDS/STONEES criteria, use of systemic antibiotics, and overall procedural tolerability and safety.

Wounds were evaluated weekly during treatment and at 1-, 3-, and 6-month follow-up visits to confirm healing stability and detect possible relapses. The study endpoints are summarized in Table 2.

### 2.6. The Protocol

#### 2.6.1. Pretreatment and NPWT Bridge

Pretreatment and NPWT bridge. The lesion is pretreated with an HA+AA gel or cream (as detailed in the SOP) to induce moisture-driven, osmotic gradient-mediated autolysis of necrotic and non-vital tissue while stimulating early neogranulation in viable tissue. After 4–5 days, if necessary, NPWT is applied to support autolytic debridement, exudate control, and granulation as a bridge therapy; otherwise, treatment continues with the cream. This step refines the wound bed and facilitates removal of non-vital tissue from walls, base, and undermining tracts, also thanks to the preliminary gel action. NPWT is leveraged to compact fragile neogranulation (microdeformation) and increase its consistency prior to infiltration.

#### 2.6.2. Pre-Infiltration Mapping

The lesion is then examined with particular attention to pediatric tissue fragility and lesion complexity. All areas of tissue deficit and undermining are mapped-these define the injection depots to accelerate healing via matrix protein biosynthesis and collagen production, following spacing and depth parameters in the SOP.

#### 2.6.3. Mechanistic Rationale

At the molecular level, injectable HA+AA has been associated with up-regulation of TGF-β1 and anti-inflammatory cytokines (IL-10), promoting resolution of chronic inflammation, fibroblast activation, and collagen/elastin synthesis. Concurrently, increased local VEGF supports neoangiogenesis and granulation, while endothelial eNOS-derived nitric oxide (NO) contributes to antimicrobial action and apoptosis of chronic inflammatory cells [14]. These converging pathways underpin the clinical effects observed-matrix deposition, granulation compaction, and accelerated re-epithelialization [7,14,15,24].

#### 2.6.4. Ten-Points Operating Checklist

Before proceeding with the injection, ten key points must be followed: (1) stressless; (2) clean cleanse; (3) disinfection; (4) syringe and needles; (5) accurate preparation; (6) body position; (7) moving the wound; (8) mapping; (9) avoid bleeding; (10) waiting time.

In full details:

1. *Stressless*. Minimize procedure-related stress and pain through caregiver/patient counseling and stepwise measures adopted by the clinical team: (a) active distraction in all needle procedures; (b) topical anesthesia (cream or flush dispersion) when needed; (c) deep sedation only in selected cases (severe agitation, autism spectrum disorder, complex comorbidities). Intranasal agents were used occasionally at the team’s discretion [25].
2. *Clean cleanse*. Gently cleanse the wound bed using non-ionic, no-rinse solutions (ozonated oils or surfactants), avoiding anionic or cationic products [22].
3. *Disinfection*. Disinfect periwound skin with 2% chlorhexidine and the wound and periwound edges with PHMB; maintain 10 min contact time. Apply with gentle, non-traumatic sponges [22].
4. *Syringe and needles*. Prepare two needles (19 G for aspiration; 21–23 G for inoculation). Keep needles out of the child’s sight; use distraction to reduce procedural discomfort.
5. *Accurate preparation*. Expose the lesion in full: assess it as a volume (ragged walls, thick base, possible fistulous tracts) rather than a simple area and perimeter.
6. *Body position*. Choose the most comfortable position close to a parent or caregiver (often in-arms). Use cartoons or music therapy; in newborns, soothing light or color stimulation can be considered.
7. *Moving the wound*. Gently tension the periwound to visualize undermining and areas of greater tissue loss or necrosis.
8. *Mapping*. Mark undermined and poorly granulating areas with a dermographic pencil to define depots (to spacing depth see SOP).
9. *Avoid bleeding*. Under magnification (microsurgical loupes), avoid vascular injury and fragile early granulation. Areas of tissue with less granulation are selected first, and if bleeding occurs, moderate compression is applied (sometimes gauze compresses can be soaked in hydrogen peroxide and left on for 15–20 s). Significant bleeding has never been observed.
10. *Waiting time*. Allow a few seconds between sequential depots; withdraw the needle fully and re-enter gently for each inoculation.
  *Analgesia and topical anesthesia*. The entire procedure can be performed after taking oral paracetamol 30 min beforehand and using a topical lidocaine hydrochloride spray or cream, covering it with a polyurethane film and waiting at least 15–20 min.
  *Documentation and consent*. Injections of HA+6AA formulations are administered clockwise or counterclockwise, and each injection must be well documented for subsequent applications, using photographs taken after obtaining written consent from the parents or caregivers (see Ethical considerations).

**Figure 1:**
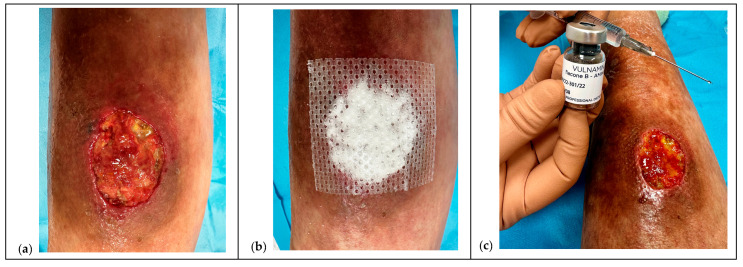

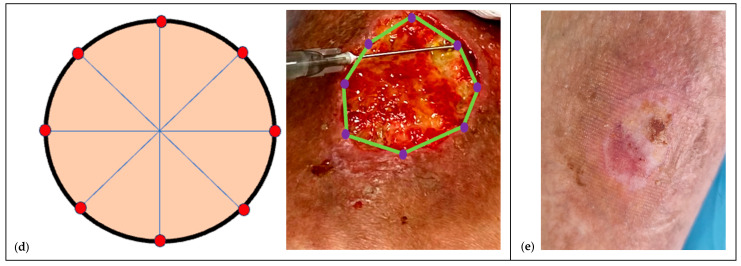
Sequential steps of the protocol with Hyaluronic Acid and Amino Acids (HA+AA) formulations in adolescent affected by Hypocomplementemic urticarial vasculitis syndrome (HUVS) (Case 15). (**a**) The lesion on initial examination shows undermining, abundant superficial exudate, areas of yellow necrosis, and fractures on the surface of the wound bed. (**b**) Application of HA+AA powder to start managing the exudate and at the same time produce an initial regeneration-repair of the tissue by inducing neogranulation. (**c**) Preparing of infiltration with injectable Hyaluronic Acid and six Amino Acids (HA+6AA) using a sterile syringe as reported in “Ten points operating checklist”. (**d**) Injection phase with HA+6AA: depots are delivered in small quantities following a counterclockwise (or clockwise) rotation along the wound edges and undermined areas. The technique requires gentle movements of the needle, progressive mapping around neogranulation “buttons,” and careful control of bleeding. The purple dots indicate the injection site, with an octagonal pattern, as highlighted by the green lines, along the edge of wound. If bleeding occurs, compression is applied for about 2 min before resuming. (**e**) Final image of the lesion, showing complete re-epithelialization, absence of exudate and signs of hypertrophic or keloid scarring, with overall healing of 100%.

**Figure 2:**
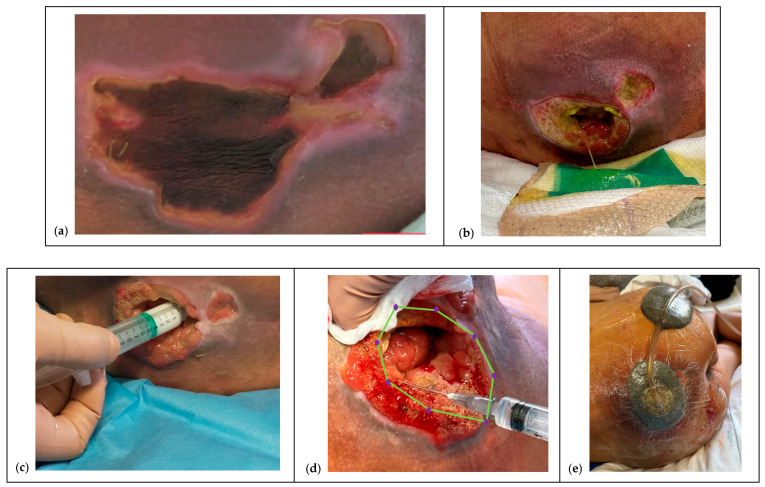

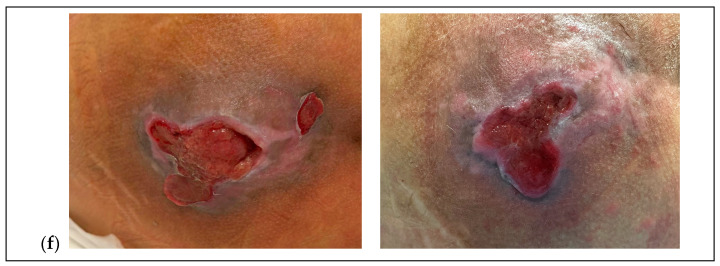
Illustrative sequence of the therapeutic protocol with Hyaluronic Acid and Amino Acids (HA+AA) in complex Pressure ulcer (PU) in pediatric patient (Case 1). (**a**) Unstageable PUs pretreated with HA+AA gel applied over necrotic tissue to induce hyperhydration, stimulate autolysis, and at the same time promote early granulation in viable areas. (**b**) When indicated, this step can be coupled with Dialkylcarbamoyl Chloride (DACC) dressings to control superficial critical colonization. (**c**) Application of HA+AA cream to enhance hydration and provide extracellular matrix support. At this stage, careful evaluation of the wound cavity is performed, with assessment of granulating tissue, periwound protection, and tissue color check to guide subsequent infiltration. (**d**) Injection phase with Hyaluronic Acid and six Amino Acids (HA+6AA): depots are delivered in small quantities following a counterclockwise (or clockwise) rotation along the wound edges and undermined areas. The purple dots indicate the injection site, with an octagonal pattern, as highlighted by the green lines, along the edge of wound. The technique requires gentle movements of the needle, progressive mapping around neogranulation “buttons,” and careful control of bleeding. If bleeding occurs, compression is applied for about 2 min before resuming. (**e**) Use of Negative Pressure Wound Therapy (NPWT) as a complementary tool to stabilize fragile granulation tissue, compact neotissue, and control exudate prior to infiltration. (**f**) Last image of the sacral PU, showing the healing of the perilesional tissue, re-epithelialization now advanced and equal to 90% of the total surface and the absence of exuberant, hypertrophic, keloid scars and cicatricial retractions with sometimes evident tissue distortions.

To facilitate reproducibility, the protocol was also schematized through illustrative diagrams, which summarize the sequence of pre-treatment, mapping, infiltration, and follow-up phases (Figure 1 and Figure 2).

### 2.7. Statistical Analysis

All collected data were analyzed descriptively, consistent with the limited sample size and the observational design of the study. Continuous variables (age, wound duration, number of treatment sessions, time to complete re-epithelialization, and follow-up length) were summarized as medians with interquartile ranges (IQR). Categorical variables (sex, wound etiology, location, stage, comorbidities, and use of co-interventions such as NPWT or DACC dressings) were reported as absolute frequencies and percentages.

The primary outcome, defined as time to complete re-epithelialization, was described using median and IQR values. Healing trajectories were visualized with Kaplan–Meier survival analysis, censoring the patient who did not achieve complete healing during follow-up.

Secondary outcomes (pain intensity, local infection, critical colonization, and tolerability) were analyzed descriptively. Pain was assessed using age-appropriate validated scales (FLACC, INRS, VAS) and qualitatively summarized, highlighting an overall reduction after the first two weeks of treatment, except for two cases requiring additional analgesic support. Local infection and critical colonization were evaluated according to the NERDS/STONEES criteria and managed with DACC dressings in three patients. Tolerability and safety were described qualitatively: no major adverse events occurred, procedural discomfort was minimal and managed with paracetamol or topical anesthesia, and two patients developed mild hypertrophic scarring, successfully treated with silicone gel sheeting.

Data were captured and managed using the institutional instance of REDCap, with role-based access control, audit trails, and pseudonymized study IDs. Datasets were exported in comma-separated values (CSV) format for analysis. All analyses and figures were generated using R (version 4.5.1, released 13 June 2025) using base and stats functions for descriptive summaries, the survival package for Kaplan–Meier estimation, and ggplot2/survminer for graphical rendering and risk-table display. Scripted workflows ensured full reproducibility of the analyses.

## 3. Results

Fifteen pediatric patients (aged 4–16 years) were enrolled (Table 1). The cohort included different wound etiologies, namely PUs (*n* = 6, 40.0%), LLU (*n* = 4, 26.7%), surgical dehiscence (*n* = 3, 20.0%), and traumatic wounds (*n* = 2, 13.3%). Most lesions were located over the sacral or gluteal area, lower limbs, and trunk, with stages ranging from III to IV according to the NPUAP/EPUAP classification.

Local infection and critical colonization were observed in three cases (20%), all managed successfully with DACC dressings without systemic complications. Increased exudate was reported in five patients (33.3%) during the early treatment phase and progressively decreased after the second week. Systemic antibiotics were required in four patients (26.7%), mainly due to secondary wound infection or peri-lesional cellulitis. No cases of sepsis or hospitalization occurred during follow-up.

The median age was 11 years [IQR 4–16], and the median wound duration prior to enrollment was 8 months [IQR 6–12]. All lesions had been previously treated in other centers with one of four previously used therapeutic regimens as co-interventions, without achieving complete healing. At the time of inclusion, wounds were staged according to EPUAP/NPIAP/PPPIA guidelines 2025 1st edition and were characterized by chronicity, pain (assessed with age-appropriate scales: FLACC, VAS, INRS), frequent comorbidities, and in several cases psychomotor impairment. Baseline demographic and clinical characteristics are summarized in Table 3.

All patients received the standardized wound hygiene protocol and HA+6AA injectable treatment, combined with specific co-interventions according to predefined criteria. Eleven patients (73.3%) required NPWT as bridge therapy (2–4 dressing changes), while 3 patients (20.0%) received DACC dressings for superficial critical colonization, and none required HA+AA gel or cream pretreatment. The percentage of patients receiving each co-intervention (NPWT, DACC, HA+AA gel/cream) and the timing of their use in relation to the injectable treatment are shown in Table 4.

A detailed per-patient dataset, including wound characteristics, treatment time-line, number of sessions and follow-up outcomes, is available in the Supplementary Materials (Table S1).

Eleven of 15 patients underwent continuous negative pressure therapy at a pressure of 75 mmHg on the first day, then increased from the second day onwards to 100 mmHg. The primary indications for use were heavy exudate, severe pain at onset, stage IV, and stage III wounds with more extensive undermining. NPWT was used for a maximum of three consecutive changes every four days and applied 30 min after the injections, thus simultaneously. In three cases where slough, bad odor, and extensive undermining were present, a DACC liner was also used for the first two applications.

The median time to complete re-epithelialization was 6 [5–12] weeks, with 14 of 15 patients (93.3%) achieving full healing.

**Figure 3:**
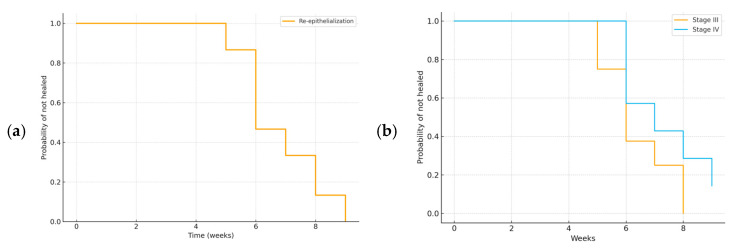
(**a**) is reported the Kaplan–Meier curve for time to complete re-epithelialization in the overall cohort. (**b**), instead, is reported the Kaplan–Meier curves stratified by wound stage (III vs. IV).

Notably, wounds achieving complete re-epithelialization within 21 days were predominantly superficial partial-thickness lesions, generally characterized by limited tissue loss and no surgical exposure. These cases displayed faster healing dynamics compared with deeper or undermined wounds, as discussed in the following paragraph, as shown by the Kaplan–Meier survival curve (Figure 3a). Kaplan–Meier time-to-event analysis confirmed a progressive increase in the cumulative probability of complete re-epithelialization, with 50% of patients healed by week 6 and over 90% by week 12, while one patient remained censored at the end of follow-up. The corresponding risk table, indicating the number of patients at risk per week, is presented in Table 5. Stratification by wound stage demonstrated a slower healing trajectory in stage IV compared with stage III lesions (Figure 3b).

Two cases showed a tendency to relapse during follow-up, one in a patient with myelomeningocele and the other in a boy with trisomy 21.

The interval between sessions was approximately 7 ± 2 days, with a median of 6 HA+6AA injection sessions [IQR 5–12]. The median follow-up period was 14 months [IQR 8–24], during which no new wound breakdowns were reported. This condition was effectively stabilized through an intensified prevention bundle that included the adoption of the Double Protection Strategy during rehoming, reassessment of wheelchair seating in orthopedic clinics, more consistent and thorough nursing by natural and social caregivers, and close liaison with local nursing staff. An additional cornerstone was the integration of occupational therapy with active and passive physiotherapy, which contributed to maintaining tissue stability without recurrences or perilesional damage.

Procedural tolerability was favorable: no significant pain was reported, as all children and adolescents received oral paracetamol before infiltration and, when required, topical anesthesia. The FLACC (Face, Legs, Activity, Cry, Consolability) scale, the VAS (Visual Analogue Scale), and the INRS (Individualized Numeric Rating Scale) were used according to patient age. In all patients, pain subsided after the first two weeks of therapy and therefore after the first two injections. The only exceptions were two cases, one with cerebral tuberculosis and one with severe autism, in whom pain persisted almost until the end of treatment, partly due to sensory hypersensitivity and difficulty in expressing needs. No major adverse events were observed during treatment or follow-up, and no sessions had to be interrupted due to distress or bleeding. In two cases, we found a mild hypertrophic scar: in the first, it was due to repeated treatments performed at other hospitals, which created intralesional scar tissue with almost no subcutaneous tissue (case 6 in Table 5). The second case was a post-traumatic, infected foot lesion (case 10), which was intensely painful upon admission and presented serious difficulties in applying dressings. The pain caused the dressings to dislodge and require reapplying, leading to the adoption of NPWT. These two cases benefited from cold atmospheric plasma (CAP) therapy, which resolved the hypertrophic scarring after 4 applications of 6 sessions each.

Overall, this exploratory case series demonstrated high healing rates and good tolerability of injectable HA+6AA in pediatric hard-to-heal wounds. Fourteen of fifteen patients achieved complete closure, with a median time to re-epithelialization of 6 weeks. Procedural tolerability was excellent, and no major complications occurred. These results support the feasibility and safety of this therapeutic approach in a fragile pediatric population.

## 4. Discussion

This prospective case series provides preliminary evidence that the use of an injectable HA+6AA, combined with standardized wound hygiene and selected co-interventions, may represent a promising therapeutic option, although the descriptive case series design without a control group limits the strength of inference. In our cohort, 14 out of 15 patients (93.3%) achieved complete healing, with a median time to re-epithelialization of 6 weeks, despite having previously undergone multiple unsuccessful treatment regimens.

Of note, two patients showed a tendency to relapse, one with myelomeningocele and one with trisomy 21. In both cases, recurrence risk was influenced by underlying factors such as cicatricial adhesions, severe motor disability, nutritional impairment, and limited social support. These findings suggest that long-term stability requires not only wound closure but also preventive strategies including rehabilitation, physiotherapy, and tailored multidisciplinary follow-up [26].

Importantly, the therapy was well tolerated, with only minimal procedural discomfort and no serious adverse events, even in a population characterized by young age, comorbidities, and fragile skin integrity. These results suggest the feasibility and apparent safety of HA+6AA injections in complex pediatric wounds, but further comparative studies are required to confirm these preliminary observations.

The clinical benefits observed are consistent with the established biological properties of HA and AA, including hydration, modulation of inflammation, fibroblast activation, and provision of substrates for collagen and elastin synthesis. These mechanisms, already outlined in the Introduction, may explain the favorable healing trajectories observed in our pediatric cohort [14].

Our therapeutic strategy incorporated wound bed preparation principles and the TIMERS framework, emphasizing tissue debridement, infection control, moisture balance, and edge advancement [6]. The wound hygiene phase preceding injection allowed selective autolysis and optimal wound bed preparation, facilitating subsequent HA+6AA infiltration.

NPWT, applied in 73.3% of cases, facilitated debridement and compaction of fragile granulation tissue [27], while DACC dressings were used effectively in cases of superficial critical colonization [28,29] according to IWII NERDS/STONEES criteria [23]. This multimodal and dynamic approach allowed us to adapt treatment to the evolving wound environment, an essential requirement in pediatric patients whose skin is particularly fragile and whose healing process differs from that of adults.

In adult populations, randomized controlled trials have shown that topical HA accelerates re-epithelialization and improves scar quality in chronic ulcers and surgical wounds [14,30]. Pediatric evidence remains scarce, but small series have reported favorable outcomes with topical HA+AA gel in burns and PUs [12,14]. However, to our knowledge, no prior studies have evaluated the injectable HA+AA complex in children and adolescents. To our knowledge, this is the first pediatric case series to investigate injectable HA+6AA within a standardized wound-care protocol. Only one pilot trial in adults with venous leg ulcers suggested improved healing trajectories with intradermal HA injections compared to standard care [31]. This case series therefore provides the first prospective pediatric data suggesting feasibility of this approach, although larger comparative trials are required.

The outcomes of our series are consistent with previous evidence on HA-based dressings and topical HA+6AA formulations, which have been shown to accelerate re-epithelialization, improve scar quality, and reduce infection risk in both adult and pediatric populations. The findings extend these observations to the use of injectable HA+6AA, suggesting that the direct delivery of HA and AA substrates into the wound bed may enhance tissue repair by simultaneously modulating inflammation, stimulating fibroblast and endothelial activity, and supporting collagen and elastin synthesis. This mechanism may contribute to restoring chronic or recalcitrant wounds to a more physiological healing trajectory [14,24,32].

When compared with other wound-healing strategies used in pediatric practice, such as negative pressure wound therapy (NPWT), biological or bioengineered dressings, and growth factor-based topical treatments, injectable HA+6AA appears to provide complementary benefits. While NPWT remains the gold standard for managing heavily exuding or undermined wounds, and bio-dressings or growth factors may accelerate epithelialization, injectable HA+6AA provides a unique means to directly modulate the extracellular matrix microenvironment through hydration, amino acid supply, and fibroblast activation. This combination could potentially shorten healing trajectories and reduce recurrence when integrated within standardized wound hygiene protocols.

In conclusion, the use of an injectable HA+6AA complex, within a multidisciplinary and structured wound care protocol, appears to be a safe and feasible approach to promote healing in pediatric hard-to-heal wounds. By addressing key elements of wound repair—hydration, control of inflammation, stimulation of fibroblasts, and provision of essential substrates for ECM synthesis—this strategy may offer an innovative contribution to improving outcomes and quality of life for pediatric patients with complex wounds and their families. Beyond biological plausibility, the favorable tolerability profile of HA+6AA injections suggests potential practical advantages, including reduced procedural pain, shorter hospitalization, and alleviation of caregiver burden, aspects of particular relevance in pediatric wound management [33,34].

*Limitations*. This exploratory study presents several limitations that should be considered when interpreting the findings. The small sample size (*n* = 15) and descriptive design limit statistical power and generalizability, preventing any firm conclusions regarding efficacy. The absence of a control or comparator group precludes causal inference and makes it impossible to quantify the independent contribution of the injectable hyaluronic acid–amino acid complex. The concomitant use of additional therapies—particularly NPWT in 73% and DACC dressings in 20% of cases—may have acted as confounding factors influencing outcomes. Furthermore, the single center setting and heterogeneity of wound etiologies may reduce external validity and applicability to other pediatric populations or clinical environments. These limitations indicate that the present results should be viewed as preliminary and hypothesis-generating rather than confirmatory, underscoring the need for adequately powered multicenter comparative trials to validate these early observations.

## 5. Conclusions

In this prospective, exploratory case series of 15 children and adolescents with hard-to-heal wounds, injectable HA+AA integrated into a standardized wound-care pathway was feasible, well tolerated, and associated with high healing rates (93.3%) and a median time to re-epithelialization of 6 weeks [IQR 5–12]. Given the frequent use of co-interventions—especially NPWT—these findings should be interpreted as hypothesis-generating rather than proof of efficacy. Future multicenter controlled studies are needed to quantify the independent effect of HA+AA injections, define optimal indications (undermining, stalled edges, tissue loss), and refine dosing and scheduling in the pediatric population.

## Figures and Tables

**Table 1:** Inclusions and exclusions criteria. When multiple wounds were present in the same patient, an index wound was selected for analysis to avoid clustering; all outcomes were analyzed per patient. Abbreviations: HA+AA: Hyaluronic Acid and Amino Acids.

Inclusion Criteria	Exclusion Criteria
Age between 4 and 16 years	Presence of uncontrolled systemic or local acute infection requiring urgent surgical intervention
Presence of at least one hard-to-heal wound (defined as <30% reduction in area at 4 weeks and non-healing >6 up to 12 months from onset)	Severe immunosuppression or ongoing chemotherapy/radiotherapy incompatible with the protocol
Wound staged according to EPUAP/NPIAP/PPPIA guidelines (2025) [17]	Uncorrected coagulation disorders or conditions contraindicating infiltration therapy
Availability of written informed consent from parents/legal guardians and assent from children and adolescents when applicable	Inability to obtain informed consent/assent. Concerns about the family’s ability to complete the outpatient protocol due to logistical constraints.
Presence of congenital or acquired chronic conditions (neurological or metabolic disorders)	Known hypersensitivity to HA+AA

**Table 2:** Study outcomes and operational definitions. Abbreviations: FLACC: Face, Legs, Activity, Cry, Consolability scale. VAS: Visual Analogue Scale. INRS: Individualized Numeric Rating Scale. IWII: International Wound Infection Institute. NERDS/STONEES: Non-healing, Exudate, Red friable tissue, Debris, Smell/ Size increasing, Temperature, Os, New areas of breakdown, Exudate, Erythema/Edema, Smell.

Outcome	Measurement Method	Timing of Assessment
**Primary outcome**	Time to complete re-epithelialization: defined as 100% epithelial coverage of the wound without exudate, confirmed at two consecutive assessments at least 7–14 days apart.	Weekly during treatment; at 1-, 3-, and 6-month follow-up
**Percentage area reduction at 4 weeks**	Relative reduction in wound surface area compared to baseline (measured by standardized digital photography with calibration and planimetry).	Baseline and week 4
**Time to 50% wound area reduction**	Number of weeks required to achieve a 50% reduction in wound area compared to baseline.	Weekly until endpoint
**Pain intensity**	Measured with age-appropriate validated scales: FLACC (<7 years), INRS (cognitively impaired), VAS 0–10 (≥7 years).	Baseline; before each injection session; at follow-up
**Exudate amount and odor**	Assessed using a 0–3 ordinal scale (0 = absent, 3 = abundant/severe).	At each dressing/injection change
**Local wound infection**	Evaluated according to IWII NERDS/STONEES clinical criteria.	At each assessment
**Use of systemic antibiotics**	Recorded if systemic antimicrobial therapy was required during treatment.	Throughout treatment and follow-up
**Tolerability and adverse events**	Documented presence of procedural pain, local bleeding requiring intervention, infection exacerbation, nodule formation, or systemic/local hypersensitivity reactions.	During and after each injection session

**Table 3:** Clinical characteristics of enrolled patients (*n* = 15). Continuous variables are expressed as median [IQR]; categorical variables as n (%). Abbreviations: PUs: Pressure ulcers. LLU: Lower limb ulcers.

Variable	Result
Age (years)	11 [6.5–16]
Sex	M 8 (53.3%), F 7 (46.7%)
Wound duration (months)	8 [6–12]
Etiology	PUs 6 (40.0%) LLU 4 (26.7%) Surgical dehiscence 3 (20.0%) Traumatic wounds 2 (13.3%)
Comorbidities	Neurological 5 (33.3%) Genetic/syndromic 3 (20.0%) Autoimmune 2 (13.3%) Others 5 (33.3%)
Wound location	Sacral/Gluteal region 6 (40.0%) LLU 7 (46.7%) Trunk/Others 2 (13.3%)
Stage	III 8 (53.3%) IV 6 (40.0%) Mixed 1 (6.7%)

**Table 4:** %: percentage of cases presenting a specific characteristic relative to the total. n/N: number of cases out of the total number. Abbreviations: IQR = Interquartile Range, representing data variability. NPWT: Negative Pressure Wound Therapy. DACC: Dialkylcarbamoyl Chloride. HA+AA: Hyaluronic Acid and Amino Acids. IWII: International Wound Infection Institute. NERDS: Non-healing, Exudate, Red friable tissue, Debris, Smell. STONEES: Size increasing, Temperature, Os, New areas of breakdown, Exudate, Erythema/Edema, Smell.

Co-Intervention	Patients Receiving n/N (%)	Typical Timing of Use
NPWT	11/15 (73.3)	Before and during injections (2–4 dressing changes) in cases of extensive tissue loss
DACC dressing	3/15 (20.0)	Before injections in wounds with local signs of critical colonization (IWII stage 1–2 according to NERDS/STONEES) *
HA+AA gel/cream	0/15 (0.0)	Pretreatment phase to induce osmotic debridement prior to infiltration

Co-interventions used in addition to HA+AA injectable treatment. * Clinical signs of superficial/critical colonization according to the IWII NERDS/STONEES criteria (stage 1–2). In this cohort, no lesions required HA+AA gel/cream pretreatment; the step is retained in the protocol for cases with devitalized tissue.

**Table 5:** Number of patients at risk per week during follow-up with Hyaluronic Acid and six Amino Acids (HA+6AA) Inj.

Week	Number at Risk
0	15
1	15
2	15
3	15
4	15
5	15
6	13
7	7
8	5
9	2
10	1
11	1
12	1

## Data Availability

The datasets generated and/or analyzed during the current study are available from the corresponding author on reasonable request.

## Supplementary Materials

The following supporting information can be downloaded at: https://www.mdpi.com/article/10.3390/children12111554/s1, Table S1: Detailed per-patient data.
