# Supplementary material for: Injectable Hyaluronic Acid and Amino Acids Complex for Pediatric Hard-to-Heal Wounds: A Prospective Case Series and Therapeutic Protocol
Source: Children (Basel). 2025 Nov 17;12(11):1554. doi: 10.3390/children12111554 (PMC12651859; doi:10.3390/children12111554)
Supplement: Supplementary file 1 [file children-12-01554-s001.zip › children-3928429-supplementary.pdf]

Patient (Age/ Gender)	Comorbidities	Wound type	Body site	Stage	Infection / Pre-treatment	HA+AA Treatment (Inj., weeks, outcome)	Other Treatments	Follow-up	Outcomes (scar, relapse, retraction)	Notes (standardized)
1 (16, M)	Paraparesis	PU	Sacral	IV	Yes / Yes	6 inj., 6 wks, Healing: full	DACC, NPWT	18 mos	Scar: regular, Relapse: none, Retraction: none	ICU; prolonged immobility
2 (14, M)	Chronic S. Dehiscence	S. dehiscence	Left lower limb	III	Yes / None	8 inj., 7 wks, Healing: full	None	18 mos	Scar: regular, Relapse: none, Retraction: none	Pseudo-lymphoma surgery
3 (6, F)	ALS	PU	Gluteal	IV	Yes / Yes	6 inj., 6 wks, Healing: full	NPWT	12 mos	Scar: regular, Relapse: none, Retraction: none	Hereditary disease
4 (10, F)	McDuffie S.	LLU	Right lower limb	III	Yes / Yes	6 inj., 6 wks, Healing: full	HA+AA	8 mos	Scar: dark, Relapse: none, Retraction: none	Hypocomplementemic syndrome; waiting LTx
5 (5, M)	Morphea	LLU	Left lower limb	III	Yes / None	9 inj., 8 wks, Healing: full	None	14 mos	Scar: dark, Relapse: none, Retraction: yes	Pansclerotic morphea
6 (6, M)	Myelomeningocele	S. dehiscence	Back	IV	Yes / None	6 inj., 6 wks, Healing: full	NPWT	24 mos	Scar: mild hypertrophy, Relapse tendency	Prenatal MMC; bladder dysfunction
7 (12, M)	Crush syndrome	Traumatic wound	Right lower limb	IV	Yes / Yes	8 inj., 7 wks, Healing: full	DACC, NPWT	12 mos	Scar: regular, Relapse: none, Retraction: none	Road accident
8 (4, F)	Myelomeningocele	S. dehiscence	Sacral	III	Yes / Yes	5 inj., 5 wks, Healing: full	NPWT	10 mos	Scar: regular, Relapse: none, Retraction: mild	Similar to case 6
9 (10, F)	Cerebral + pulmonary TB	PU	Sacral	IV	Yes / Yes	10 inj., 9 wks, Healing: full	NPWT	12 mos	Scar: dark, Relapse: none, Retraction: mild	ICU; active infection
10 (7, F)	Autism	Traumatic wound	Left foot	III	Yes / Yes	6 inj., 6 wks, Healing: full	DACC, NPWT	18 mos	Scar: mild hypertrophy, Relapse: none, Retraction: none	Sensory hypersensitivity; CAP therapy

11 (14, M)	Trisomy 21	PU	Trochanteric	IV	Yes / Yes	9 inj., 8 wks, Healing: full	NPWT	12 mos	Scar: regular, Relapse: tendency, Retraction: none	Severe motor disability; poor mobility
12 (13, M)	Crouzon syndrome	PU	Sacral	III	No / None	6 inj., 6 wks, Healing: full	NPWT	14 mos	Scar: regular, Relapse: none, Retraction: none	ICU; immobility
13 (11, F)	Respiratory Distress Syndrome	PU	Gluteal	III	No / Yes	6 inj., 5 wks, Healing: full	None	12 mos	Scar: regular, Relapse: none, Retraction: none	Brain hemorrhage; CTICU
14 (14, M)	LAD 1	LLU	Bilateral lower limbs	III	Yes / Yes	10 inj., 8 wks, Healing: full	NPWT	24 mos	Scar: regular, Relapse: none, Retraction: none	Soft tissue loss; biologics
15 (16, F)	LAD 1	LLU	Bilateral lower limbs	IV/III	Yes / Yes	12 inj., 8 wks, Healing: full	NPWT	16 mos	Scar: regular, Relapse: none, Retraction: none	BMT; soft tissue loss

Table S1. Detailed per-patient clinical dataset of the 15 pediatric cases treated with injectable Hyaluronic Acid and Amino Acids (HA+AA). The table reports demographic variables, comorbidities, wound etiology and staging, infection status, pre-treatments and co-interventions, number of HA+AA sessions, healing time, follow-up duration, scar outcomes, relapse tendency, and standardized clinical notes. Data are presented as recorded in the prospective case series. Abbrevations: PU: Pressure Ulcer; S. Dehiscence: Surgical Dehiscence; ALS: Amyotrophic Lateral Sclerosis; McDuffie S.: McDuffie Syndrome; TB: Tuberculosis; MMC: Myelomeningocele; LAD 1: Leukocyte Adhesion Deficiency type 1; BMT: Bone Marrow Transplant; CAP therapy: Cold Atmospheric Plasma therapy; HA+AA: Hyaluronic Acid and Amino Acids; Inj.: Injection(s); NPWT: Negative Pressure Wound Therapy; DACC: Dialkylcarbamoyl Chloride; ICU: Intensive Care Unit; CTICU: Cardiothoracic Intensive Care Unit; wks: weeks; mos: months.
